# HIV-1-Induced Small T Cell Syncytia Can Transfer Virus Particles to Target Cells through Transient Contacts

**DOI:** 10.3390/v7122959

**Published:** 2015-12-12

**Authors:** Menelaos Symeonides, Thomas T. Murooka, Lauren N. Bellfy, Nathan H. Roy, Thorsten R. Mempel, Markus Thali

**Affiliations:** 1 Graduate Program in Cell and Molecular Biology, University of Vermont, Burlington, VT 05405, USA; msymeoni@uvm.edu (M.S.); royn@email.chop.edu (N.H.R.); 2 Department of Microbiology and Molecular Genetics, University of Vermont, Burlington, VT 05405, USA; lbellfy@uvm.edu; 3 Departments of Immunology and Medical Microbiology, University of Manitoba, Winnipeg, MB R3E 0T5, Canada; thomas.murooka@umanitoba.ca; 4 Center for Immunology and Inflammatory Diseases, Division of Rheumatology, Allergy and Immunology, Massachusetts General Hospital, Harvard Medical School, Boston, MA 02114, USA; tmempel@mgh.harvard.edu

**Keywords:** HIV, cell-cell fusion, syncytia, humanized mouse, 3D culture, live cell imaging

## Abstract

HIV-1 Env mediates fusion of viral and target cell membranes, but it can also mediate fusion of infected (producer) and target cells, thus triggering the formation of multinucleated cells, so-called syncytia. Large, round, immobile syncytia are readily observable in cultures of HIV-1-infected T cells, but these fast growing “fusion sinks” are largely regarded as cell culture artifacts. In contrast, small HIV-1-induced syncytia were seen in the paracortex of peripheral lymph nodes and other secondary lymphoid tissue of HIV-1-positive individuals. Further, recent intravital imaging of lymph nodes in humanized mice early after their infection with HIV-1 demonstrated that a significant fraction of infected cells were highly mobile, small syncytia, suggesting that these entities contribute to virus dissemination. Here, we report that the formation of small, migratory syncytia, for which we provide further quantification in humanized mice, can be recapitulated *in vitro* if HIV-1-infected T cells are placed into 3D extracellular matrix (ECM) hydrogels rather than being kept in traditional suspension culture systems. Intriguingly, live-cell imaging in hydrogels revealed that these syncytia, similar to individual infected cells, can transiently interact with uninfected cells, leading to rapid virus transfer without cell-cell fusion. Infected cells were also observed to deposit large amounts of viral particles into the extracellular space. Altogether, these observations suggest the need to further evaluate the biological significance of small, T cell-based syncytia and to consider the possibility that these entities do indeed contribute to virus spread and pathogenesis.

## 1. Introduction

Human immunodeficiency virus type 1 (HIV-1) primarily infects CD4^+^ T lymphocytes and macrophages. Acute infection is characterized by flu-like symptoms, and is followed by a long asymptomatic period, until finally developing into profound immune deficiency resulting primarily from CD4^+^ T cell depletion. The spread of virus between infected and susceptible target cells can take place via several pathways, including: release of cell-free virus particles which are then stochastically encountered by a target cell; sequestration of virus particles by dendritic cells and subsequent delivery of these particles to a target cell (a process termed *trans*-infection [1]); and the process of cell-to-cell transmission, whereby an infected cell directly interacts with a target cell, thus forming a transient adhesion structure known as the virological synapse (VS; [2,3]), which facilitates transfer of newly released viral particles. Such interactions are thought to occur most frequently in secondary lymphoid tissue, such as in lymph nodes and the gut-associated lymphoid tissue (GALT), where high cell density and migratory scanning behavior of T cells, facilitated by the architecture of the stromal environment, provide conditions conducive to controlled cell–cell interactions, including antigen presentation through the immunological synapse [4,5,6,7].

Cell-to-cell transmission of HIV-1 has been extensively studied for more than two decades (for recent reviews: [8,9]). While *in vitro* studies have long suggested that this mode is more efficient than cell-free virus transmission [10], it remained unclear why producer cells (which express the viral envelope glycoprotein, Env) would not automatically fuse with target cells (which express the viral receptor/coreceptors) once a VS forms. However, various viral and cellular mechanisms/factors, including retrieval of Env from the surface of infected cells [11,12] and Env’s interaction with immature Gag, which is known to repress Env’s fusion activity in particles [13,14,15,16,17] and at the virological presynapse [18], have since been shown to help preserve the integrity of the VS by preventing producer-target cell fusion (for a discussion, see also [19]). Syncytia, which are multinucleated entities that form when Env-expressing (infected) cells fuse with target cells, were thus considered to be artifacts of cell culture and/or were thought to occur in infected individuals only if HIV-1-infected dendritic cells or macrophages occasionally fuse with target T cells. As will be described in the following, however, observations made in lymph nodes of HIV-1-infected humanized mice [20], together with two (largely ignored) earlier reports that documented lymphocyte-based small syncytia in secondary lymphoid tissue of infected individuals [21,22], forced us to reconsider the significance of HIV-1-induced T lymphocyte-based syncytia.

## 2. Results and Discussion

### 2.1. Quantification of HIV-1-Induced Small Syncytia in Lymph Nodes of Humanized Mice

**Figure 1:**
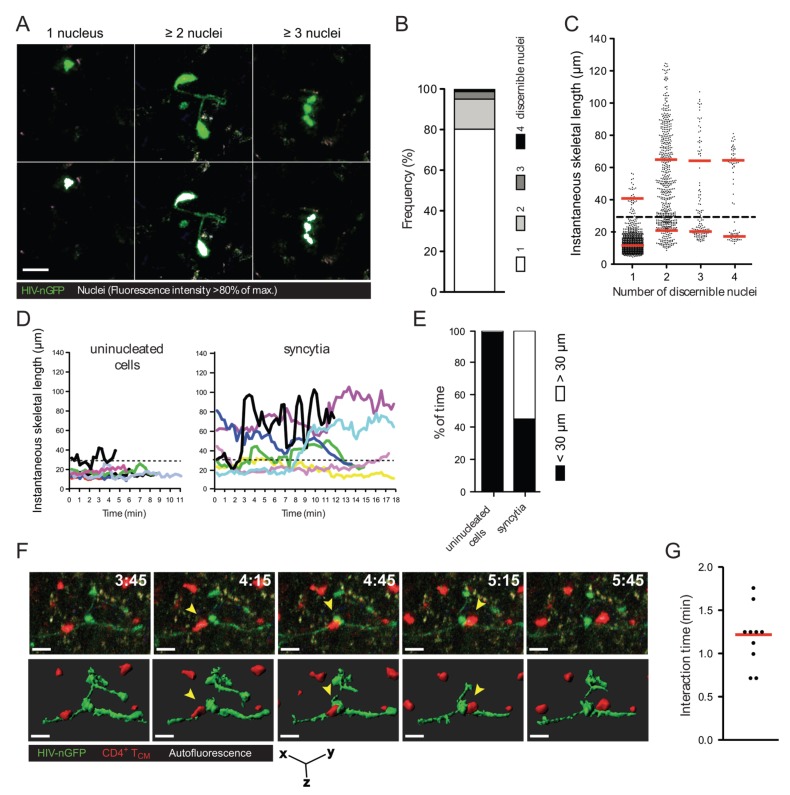
Morphology, frequency, and cellular interactions of HIV-1-induced syncytia in the lymph node. (**A**) Intravital micrographs of lymph node cells infected *in situ* with HIV-nGFP (day 2) reveal both individual infected cells and multinucleated syncytia. In the bottom panels, the nuclei of infected cells, whose location is identified by a discrete increase in fluorescence intensity, are rendered white, based on an 80% of maximum fluorescence intensity threshold; (**B**) Frequency of HIV-1-infected cells with increasing numbers of discernable nuclei; (**C**) Instantaneous skeletal lengths of individual infected cells and multinucleated syncytia. Red lines indicate means. A threshold length of 30 microns (dotted line) was used to differentiate between coordinate *vs.* uncoordinated movements. Data from 8 movies recorded in two independent experiments are shown; (**D**) Representative traces of HIV-1-infected lymph node cells showing instantaneous skeletal lengths over time; (**E**) Percent of time an individual infected cell or a multinucleated syncytium displayed coordinated and uncoordinated movements, based on a skeletal length threshold of 30 microns. Data from 8 movies recorded in two independent experiments are shown; (**F**) 3D time-lapse image sequence of a transient cellular interaction between an HIV-1-induced syncytium (HIV-nGFP; green) and an uninfected T cell (CMTMR-labeled; red) in the lymph node of BLT mice. In the bottom panel, 3D cellular surface rendering shows the transient nature of the contact. Elapsed time in min:s. Yellow arrowheads indicate the interacting uninfected T cell. Scale bar = 15 μm; (**G**) Interaction times between HIV-1-induced syncytia and uninfected T cells. Data from 2 movies (*n* = 9) is shown. Red line indicates the median.

A considerable proportion of HIV-1-infected cells in the lymph node of humanized bone marrow/liver/thymus (BLT) mice exhibit elongated morphologies and reduced migration speed. Further, multiphoton intravital microscopy (MP-IVM) revealed that, surprisingly, a large fraction of these cells were syncytia [20]. To document this finding with more granularity, the number of discernible nuclei (revealed using an HIV-1 reporter strain that expresses EGFP fused to a nuclear localization signal, referred to as HIV-nGFP; see Figure 1A and [20]) and the instantaneous skeletal length of all infected cells in the lymph node were measured. As shown in Figure 1B, ~20% of infected cells are multinucleated with two, three, or four discernible nuclei (in decreasing frequency), and we did not observe any cells with five or more discernible nuclei during our imaging studies. However, it is possible that visualizing syncytia using HIV-nGFP may underestimate the number of nuclei in syncytia, since overlapping nuclei may appear as a single nucleus in some instances. Alternatively, larger syncytia may be more susceptible to apoptosis. Nevertheless, we conclude that HIV-1-induced syncytia are numerous in the lymph node, but remain small two days post-infection despite having demonstrated fusion competence. At a later time-point, large syncytia develop occasionally [23], though they likely involve non-lymphoid cells and thus may not be purely T cell-derived.

We also sought to further quantify the unusual morphologies and behaviors adopted by HIV-1-induced (small) syncytia. As documented in Figure 1A and Movie S1, the nuclei within syncytia can be arranged either in a tightly clustered (coordinated) or in a scattered (uncoordinated) fashion, and dynamically switch between these two modes (see also [24]). This is reflected in the measurements of instantaneous skeletal length (Figure 1C), where the morphologies of syncytia with three or four nuclei appear to stratify into two groups: one of syncytia with short skeletal length (corresponding to coordinated forms, and resembling uninucleated cells), and one of syncytia with three to four times larger skeletal length (corresponding to an uncoordinated form). Syncytia with two nuclei exhibited much more continuous range of sizes and shapes, suggesting that nuclei in these entities were better able to decouple from each other.

Despite their varied morphologies, small syncytia have one thing in common: their nuclei are often not situated in close proximity (within a few microns) to each other (see also Figure 1D–E for further quantification). Consequently, if such entities were to form in the lymph nodes of HIV-1-infected individuals, most of them would not be detected using methods typically used to visualize infected cells in lymphoid tissues, such as immunocytochemistry of thin (three to five micron) sections. Under these conditions, only small syncytia that happen to be in the plane of the tissue slice and in a small compact shape when the tissue is fixed would become discernable as syncytia. Thus, it is possible that small HIV-1-induced syncytia are much more frequent within infected lymphoid tissues than previously thought [21,22].

Due to the high prevalence of small syncytia (two to four nuclei, but not larger) in the lymph node, we asked whether they continue to undergo fusion with target cells, or rather, if they can transiently contact uninfected T cells (*i.e.*, without fusing with them). To test this, we imaged the interactions between HIV-1-induced syncytia and adoptively transferred CellTracker Orange-labeled uninfected central memory-like T (T_CM_) cells in the lymph node of humanized mice using MP-IVM. We found that syncytia typically formed transient contacts (no longer than two minutes) with uninfected T cells that did not lead to fusion (Figure 1F and Movie S2). These transient contacts show that the observed HIV-1-induced T cell syncytia are not merely “fusion sinks” [25], as is typically observed when T cells are grown in 2D culture.

### 2.2. *In Vitro* Recapitulation of HIV-1-Induced Small T Cell Syncytia

Given the surprisingly high proportion of syncytia in the lymph node of HIV-1-infected humanized mice and, as discussed above, possibly also in HIV-1-infected individuals, we began to further investigate the formation, morphology, and behavior of HIV-1-induced syncytia within reductionist cell culture systems that permit live cell imaging at (relatively) high spatial resolution. However, syncytia grown in traditional cell culture systems, such as suspension culture, do not display the elongated morphologies that we observed in the lymph node of humanized mice ([20] and Figure 1), but rather remain rounded and can consist of dozens to hundreds of nuclei. Further, while a small series of *in vitro* studies published two decades ago showed that large T cell syncytia can display behavior that is reminiscent of that of infected individual cells [26,27,28,29], those syncytia contained thousands of nuclei and thus obviously are not representative of the small syncytia observed in lymph nodes either. As documented, however, in recent studies and as discussed in commentaries [30,31,32], T cells grown in three-dimensional (3D) cell culture systems, such as hydrogels composed of ECM, better reflect their *in situ* behaviors [6,33] compared to those grown in suspension culture, likely because distinct ECM tracks, structurally similar to the fibroreticular networks in lymph nodes, provide guidance structures for migrating lymphocytes in those *in vitro* systems [34]. We therefore started using 3D ECM hydrogels for our experiments and, as will be described in the following, immediately noticed profound differences in the appearance of syncytia, compared to syncytia observed in 2D culture.

**Figure 2:**
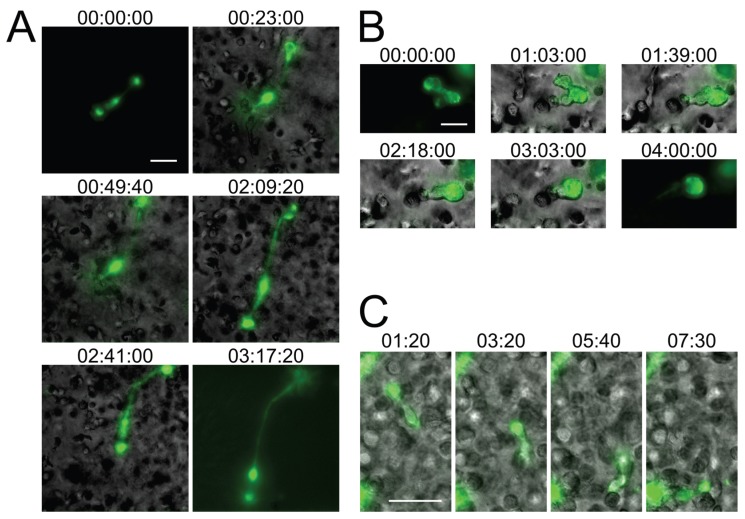
Small T cell syncytia form in HIV-1 NL4-3^Gag-iGFP^-infected 3D ECM hydrogel cultures and closely recapitulate *in situ* observations. (**A**) A syncytium with two nuclei in a collagen hydrogel culture of infected human primary CD4^+^ T cells (stills from Movie S3); (**B**) A syncytium with two nuclei in a Matrigel hydrogel culture of infected CEM-SS T cells exhibiting switching from uncoordinated to coordinated migration (stills from Movie S4); (**C**) A uninucleated infected human primary CD4^+^ T cell exhibiting directed amoeboid migration in a collagen hydrogel (stills from Movie S5). Scale bars = 30 μm. Elapsed times in h:min:s for (A,B), and in min:s for (C). Gag-iGFP signal is pseudocolored in green. Brightfield images are in shades of gray. In (A,B), the syncytium is shown without the brightfield image for the first and last stills for clarity.

When HIV-1-infected primary CD4^+^ T cells or CEM-SS T cells were cultured in 3D ECM hydrogels composed of either Matrigel or human collagen type I, the syncytia that formed were typically small (2–5 nuclei), and exhibited dynamically changing morphologies, including very elongated forms that have not been observed in 2D cultures (Figure 2A,B and Movies S3, S4 and S6–S9). The migratory behavior of infected T cells, whether uninucleated or multinucleated, in 3D cultures was also different, with cells showing directed amoeboid (non-adhesive) migration (Figure 2C and Movie S5), as already documented by others in detail for uninfected lymphocytes [31,35,36]. When quantifying the rate of syncytium formation, we also found that the number and size of syncytia tended to be reduced in 3D compared to 2D culture (data not shown), but these analyses will need to remain preliminary while we further investigate the contribution of factors that likely impact fusion, such as cell density or ECM composition. Still, during the development of 3D ECM hydrogel cultures, we already made several observations (which we do not document here, however) that we think should guide further development of this *in vitro* system. First, collagen better facilitated fast and directed amoeboid migration compared to gels composed of Matrigel, whereas cells projected large pseudopodia more frequently in Matrigel than in collagen. Second, virus release, seen as large accumulations of extracellular Gag-iGFP puncta (to be discussed later, in Section 2.4) was enhanced in Matrigel compared to collagen. Third, primary CD4^+^ T cell syncytia were more elongated than CEM-SS syncytia, but syncytia of both cell types exhibited switching behavior.

Importantly, while the current *in vitro* studies of small HIV-1-induced syncytia need to remain qualitative in nature, for the reasons outlined above, the experiments shown in Figure 2 and Movies S3–S5 nevertheless already provide additional relevant new information about HIV-1-induced syncytia: First, due to the presence in humanized BLT mice of multiple human immune cell lineages, including macrophages and dendritic cells (which can undergo fusion with HIV-1-infected T cells), we could not yet state with confidence that the observed small syncytia (in humanized mice) are resulting purely from homotypic T cell-T cell fusions, and whether this might explain differences noted between this model and the classical T cell culture system. Now, using only T cells in 3D culture, we observe very similar phenotypes, which strongly suggests that the small syncytia observed *in situ* are indeed also purely T cell-based. Second, in the 3D culture system, cells were infected with an X4-tropic HIV-1 (NL4-3^Gag-iGFP^), whereas R5-tropic virus was used in the humanized mouse experiments, demonstrating that the phenotype of these syncytia is not determined by virus tropism, but by the environment the cells experience. Third, whereas a fully replicative virus was used in the humanized mouse experiments, with or without the inclusion of nGFP to mark nuclei, in the 3D culture experiments we utilized the Gag-iGFP-tagged virus which is not fully replicative and where the nuclei are instead marked by the absence of GFP signal due to the exclusion of the marker from the nucleus. We can therefore corroborate the observations made using nGFP-tagged virus with the use of another reporter system to mark the nuclei, and show that these syncytia form during the first round of infection and likely are not a consequence of genetic drift or adaptation to the particular cells utilized in either study.

Overall, these *in vitro* experiments demonstrate that the migratory behavior and multinucleated cells closely resemble those that are found in the lymph node of humanized mice (Figure 1). Although additional modifications to this model, for instance through addition of stromal and dendritic cell populations, would further recapitulate the complex intercellular interactions observed in lymphoid tissues [37], we conclude that even a very basic 3D experimental system much better reflects the consequences of viral infections than traditional (2D) cell culture systems (see also [32]).

### 2.3. Small T Cell Syncytia Can Transfer Viral Particles to Target Cells through Transient Contacts

Thus far, syncytia have been thought of as merely cytopathic features of HIV-1 infection, exaggerated in the artificial environment of 2D cell cultures, where syncytia continue to fuse with uninfected cells [29] until they die, likely due to excess karyogamy (nuclear fusion within the syncytium) [38]. Given our data showing that syncytia actually stay small in lymphoid tissues of humanized mice (and likely in infected individuals) shortly after infection, we reasoned that they may contribute to virus dissemination by transmitting virus.

To then start addressing this question, *i.e.*, whether syncytia, specifically the small, highly mobile ones that we now can recapitulate *in vitro*, can transfer viral particles without fusing with target cells, we performed live cell time-lapse microscopy studies in the 3D ECM hydrogel system, using T cells infected with HIV-1 NL4-3^Gag-iGFP^ [39]. The GFP tag inserted between the MA and CA domains of Gag, flanked by protease sites, allows for the visualization of assembly and polarization of Gag in infected cells as well as the subsequent release of virus particles followed by transfer to uninfected target cells. Because this virus is not fully replicative, we did not seek to evaluate whether virus transfer events result in productive infection of target cells (though we have no reason to assume that they would not, if fully competent virus is utilized; see below for further discussion), but instead focused on whether we could observe contact-mediated virus transfer and subsequent separation of producer and target cell without the formation of a syncytium. Because interactions between HIV-1-infected and uninfected T cells have not previously been examined in 3D cultures *per se*, we also tracked uninucleated infected cells to study how cell-to-cell transfer occurs under conditions that more closely recapitulate their *in vivo* behaviors.

**Figure 3:**
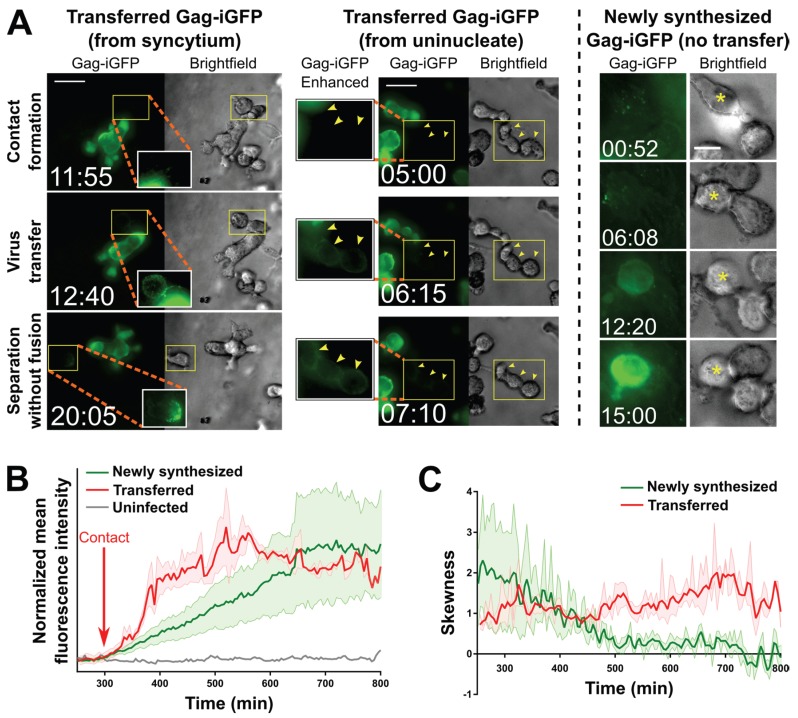
Transfer of virus particles from HIV-1 NL4-3^Gag-iGFP^-infected uninucleated CEM-SS cells and CEM-SS syncytia to uninfected CEM-SS cells in 3D ECM hydrogels. (**A**) (Left); A syncytium with 3 nuclei transfers virus to target cells, one of which then migrates away (see also Movie S7). (Middle); A uninucleated infected cell transfers virus to three target cells (yellow arrowheads; see also Movie S6). (Right): An uninfected cell with Gag-iGFP puncta on its surface (asterisk) begins developing newly synthesized Gag-iGFP signal ~12 h later, while a cell it is attached to throughout this time that did not harbor Gag-iGFP puncta is not seen to develop Gag-iGFP signal. Scale bars = 30 μm ((left) and (middle)) or 10 μm (right). Elapsed time in hours:minutes. Gag-iGFP signal is pseudocolored in green. Brightfield images are in shades of gray. To show transferred virus particles more clearly, the areas indicated by yellow boxes are shown in white-boxed enhanced insets with 1.5× enlargement and a 0.6 gamma correction applied; (**B**) Average of the mean fluorescence intensities of five target cells exhibiting either transferred Gag-iGFP (red), or five other target cells developing newly synthesized Gag-iGFP from a previously unseen infection event (green). An uninfected cell is shown in grey. The individual traces were aligned according to the time where Gag-iGFP signal began increasing (which was the time of contact with the producer cell in all cases for the transfer traces; red arrow), and the averaged curves were manually normalized to baseline and smoothed (2nd order and 2 nearest neighbors); (**C**) The average skewness of the signal across these same cells was plotted. Skewness close to zero indicates evenly distributed, or hazy signal, while higher positive skewness indicates punctate signal. Error boundaries in (B,C) represent the standard error of the mean.

Strikingly, very similar virus transfer events were observed when either uninucleated or multinucleated infected cells were viewed (Figure 3A, left and middle, and especially Movie S6 where both types of events occur almost simultaneously in the same field). In both cases, transfer of virus to target cells, which can be discerned in time-lapse movies (Movies S6–S8), is reflected in a relatively sudden increase in fluorescence intensity observed (Figure 3A, left and middle) and measured (Figure 3B, red trace) on the recipient cells. The sudden increase contrasts with a much more gradual increase that can be observed (Figure 3A, right) and measured (Figure 3B, green trace) in cells that became infected at an earlier, unseen time point, and eventually (likely 12–18 h later; [39]) started to newly synthesize Gag-iGFP as part of a productive infection at the time of imaging. Further, the two distinct types of events can be identified by the localization and nature of the Gag-iGFP signal: Firstly, upon virus transfer, material is deposited onto the surface of target cells (Figure 3A, left and middle), in contrast to newly synthesized Gag-iGFP, which appears only within the cytoplasm (Figure 3A, right). Secondly, the punctate nature of transferred Gag-iGFP (Figure 3C, red trace) is clearly distinct from the much more diffuse signal (likely corresponding to GFP that was intracellularly cleaved out of the tagged protein) seen when Gag-iGFP is newly synthesized within the cell (Figure 3C, green trace). These patterns closely match the original report of this tagged virus strain by the Chen group [39].

A defining feature of transfer events was that producer and target cells showed directed motility before or after (or even during) the moment of transfer (somewhat reminiscent of kinapses; [40]). Additionally, the Gag puncta (which we interpret to be released virus particles) transferred upon contact seemed to rapidly distribute to other uninfected cells that were already in contact with the uninfected cell closest to the infected cell (Movies S6–S7). In one case (Movie S8), the infected syncytium was seen to “drag” the previously immobile uninfected cell across the imaging field, and then “drop” it while migrating away, leaving Gag puncta on its surface. Overall, such dynamic transfer events have not previously been observed. They clearly differ from what appeared to be more static contacts between infected and uninfected cells during which virus transfer has been documented so far [39]. Most importantly though, syncytia have not previously been reported to transfer virus to target cells by contact, or even to merely be able to contact an uninfected cell without fusing with it.

### 2.4. Pools of Cell-Free Virus Particles Are Deposited by Migrating HIV-1-Infected T Cells

**Figure 4:**
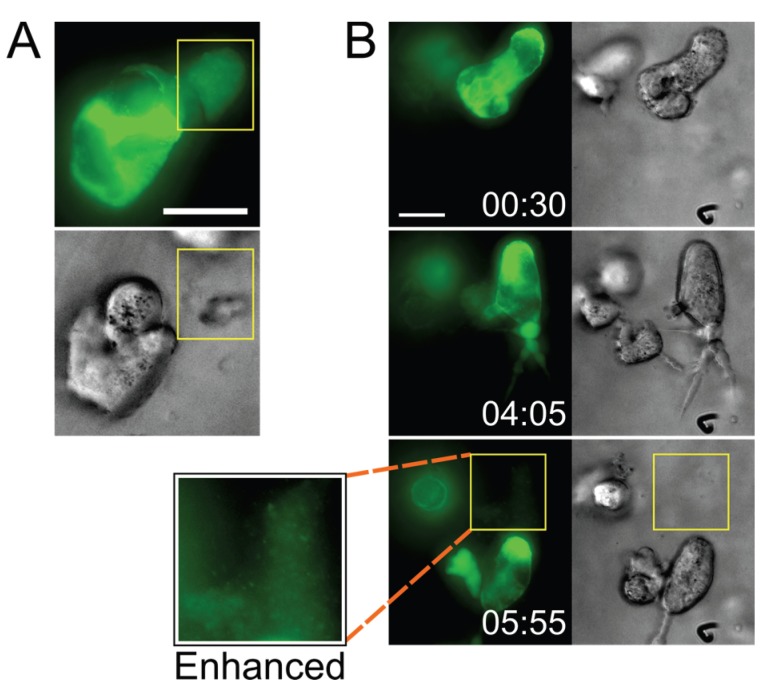
Infected cells deposit virus particles into pools within their surrounding stroma. (**A**) A CEM-SS syncytium in Matrigel culture releases a very dense accumulation of Gag-iGFP puncta into a cell-free pocket (area bounded by yellow box; still from Movie S7); (**B**) A CEM-SS syncytium in Matrigel culture migrates across the field, leaving behind a trail of dense Gag-iGFP puncta (area bounded by yellow box), shown also at 2× magnification and with brightness enhanced in a white-boxed inset (see also Movie S9). Scale bars = 20 μm. Elapsed time in h:min. Gag-iGFP signal is pseudocolored in green. Brightfield images are in shades of gray.

Intriguingly, besides observing particle transfer upon cell-cell contact, we also found distinct accumulations of densely packed virus (released by infected cells) in what appeared to be pockets that had formed within the ECM hydrogel (Figure 4 and Movie S9; also see the beginning of Movie S7). We have also observed cells depositing a trail of released Gag puncta in their wake as they migrated through the ECM (from a uninucleated cell in Movie S6, and from a syncytium in Movie S9). Although the accumulations appeared to dissipate a few minutes after they were visible (data not shown), it was not clear whether this was due to photobleaching or whether the virus particles had diffused away.

The significance of these observations (the deposition of free virus by small syncytia) remains unclear, but it would seem quite likely that potential target T cells, when they encounter such deposits, could become infected. Notably, a recent electron tomography study of the GALT documented comparable dense pools of free virions between cells (but apparently not in clefts of VSs) in HIV-1-infected humanized mice (see especially Figure S5 in [41]). Together, these observations suggest that free viral particles may contribute more to virus spread than expected based on numerous *in vitro* studies which concluded that cell-free virus is inefficiently transmitted to target cells. All those *in vitro* analyses, however, were performed in traditional systems, *i.e.*, by incubating target cells in medium containing suspended virus particles, or even with experiments where cultures of cells are (gently) shaken [42]. Obviously, pools of virus, as they can be found in our 3D system and in stroma of lymphoid tissue in humanized mice [41], would not form under those conditions, and potential target cells would not be exposed to locally concentrated virus. This may have led us to underestimate the relative contribution of cell-free HIV-1 infection to virus spread (e.g., [43,44,45]).

Of note, though, we have thus far observed these cell-free virus accumulations only in Matrigel hydrogels, and not in collagen hydrogels. However, both ECM models have limitations: Matrigel is derived from murine sarcoma cells which secrete a very complex basement membrane-like ECM that does not closely model the lymph node stroma (though it includes collagen type I), whereas the collagen utilized here was pure recombinant human collagen type I alone, which is a much more simplistic ECM that also does not accurately represent the lymph node. Therefore, we cannot conclude that one or the other model is more appropriate, and some of the events (such as the cell-free virus accumulations) may be influenced by the composition of the ECM.

### 2.5. Perspectives: A Role for Small T Cell Syncytia in HIV-1 Spread and/or Pathogenesis?

This communication establishes that small HIV-1-induced T cell syncytia, as they occur to a significant extent in lymphoid tissue of humanized mice and likely in infected individuals, can be recapitulated in simple 3D cell culture systems. It also demonstrates that such small syncytia can transfer virus to uninfected cells upon transiently contacting them, and it reveals that HIV-1-infected T cells (uninucleated and multinucleated) deposit pools of particles that likely would be able to infect cells as they migrate through those accumulations.

Given their emergence early in the infection process (as documented in humanized mice; [20] and Figure 1), it seems likely that small HIV-1-induced syncytia significantly contribute to virus spread and, equally importantly, that they contribute to pathogenesis (e.g., by triggering bystander killing through pyroptosis [46,47]. Confirming (or refuting) these hypotheses, however, necessitates extensive additional studies in humanized mice and/or the implementation of imaging techniques that allow quantitative, long-term analyses at the cell population level in 3D cultures. In conclusion, we propose that our view of what constitutes a potentially propagative or pathogenic HIV-1-infected entity should include not only uninucleated cells but also small, T cell-based syncytia.

## 3. Experimental Section

### 3.1. Cells, Plasmids, and Reagents

The following reagent was obtained through the NIH AIDS Research and Reference Reagent Program, Division of AIDS, NIAID, NIH: CEM-SS cells (Cat. #776) from Dr. Peter L. Nara [48,49,50]. CEM-SS T cells were maintained in RPMI 1640 medium supplemented with 10% FBS, 100 units/mL penicillin and 100 μg/mL streptomycin. HEK293T cells were maintained in DMEM supplemented with 10% FBS, 100 units/mL penicillin and 100 μg/mL streptomycin. All experiments were conducted in media supplemented with 10% FBS and without antibiotics.

Human primary CD4^+^ T cells were isolated from whole blood of a healthy donor by ficoll separation and subsequent negative selection (Miltenyi Biotech Cat. #130-091-155). Cells were activated with 5 μg/mL phytohemagglutinin in the presence of 50 units/mL interleukin-2 in RPMI 1640 supplemented with 10% FBS and antibiotics. After 24 h, cells were washed and cultured in the same medium containing interleukin-2 and without phytohemagglutinin. Cells were used for infections 24 to 48 h later.

The proviral plasmid NL4-3^Gag-iGFP^ [39] was obtained from Dr. Benjamin Chen (Mount Sinai School of Medicine, New York, NY, USA). An R5-tropic pIeG-nef+ reporter strain (on the NL4-3 proviral backbone) that expresses EGFP with an N-terminal nuclear localization signal (termed HIV-nGFP) was used to visualize the nuclei of syncytia *in vivo*, as previously described [20]. nGFP is enriched in all nuclei of HIV-1-induced syncytia [20], and their numbers were determined by discrete increases in GFP intensity, based on an 80% of maximum fluorescence intensity threshold.

Matrigel (Cat. #354234) was purchased from BD Biosciences (Sparks, MD, USA). Human collagen type I solution (VitroCol; Cat. #5007) was purchased from Advanced BioMatrix (San Diego, CA, USA).

### 3.2. Intravital Multiphoton Microscopy and Image Analysis

MP-IVM of HIV-1-infected cells within the popliteal lymph node of BLT mice [51] was performed as described previously [20]. A MaiTai Ti:sapphire laser (Newport/Spectra-Physics) was tuned to 920 nm for optimized excitation of the fluorescent probes used. Intravital movies (S1 and S5) are maximum intensity projections of 11 optical sections (512 × 512 pixels) with 4 μm Z-spacing, taken every 15 s to provide imaging volumes of 40 μm in depth. Imaging depth was typically confined to 80–150 μm below the lymph node capsule. 2D skeletal lengths were measured in ImageJ as the longest path connecting front and end of unbranched cells or as the sum of path lengths of all branches of multi-branched syncytia at each time frame (“instantaneous skeletal length”). The instantaneous skeletal length data shown in Figure 1C were stratified into “high” and “low” skeletal lengths using a cutoff at 30 μm based on the evident bimodal distribution of the data in order to display means, but these were not used for any statistical tests. Nuclei within infected cells (using the HIV-nGFP reporter) were identified based on a discrete increase in fluorescent intensity, rendered using an 80% of maximum fluorescent intensity threshold, and enumerated at each time frame. To visualize cell-to-cell interactions, T_CM_ cells were generated *in vitro*, labeled with CellTracker Orange (CMTMR: Molecular Probes Cat. #C2927), and adoptively transferred into the footpad of BLT mice, as previously described [20].

### 3.3. Virus Production and Infection

Viruses were produced in HEK293T cells by cotransfection of pNL4-3^Gag-iGFP^ and pVSV-G using calcium phosphate precipitation (Invitrogen, Carlsbad, CA, USA). Virus-containing supernatants collected 48 h post-transfection were cleared of cell debris by centrifugation at 2000 rcf for 10 min, filtered through a 0.4 μm filter, aliquoted, and stored at −80 °C.

To infect CEM-SS or primary CD4^+^ T cells with HIV-1 NL4-3^Gag-iGFP^, 2 × 10^6^ or 5 × 10^6^ cells (respectively) were pelleted and resuspended in 450 μL of CO_2_-independent medium (Gibco, Grand Island, NY, USA) supplemented with 10% FBS, and 10 μL of virus-containing supernatant was added (resulting in ~20% of cells being infected by the time of imaging). The cells were gently shaken in round-bottom 5 mL tubes for 2 h at 37 °C, then 3 mL of prewarmed RPMI 1640/10% FBS were added, the cells were pelleted, resuspended in 8 mL RPMI 1640/10% FBS, and incubated at 37 °C/5% CO_2_.

### 3.4. Embedding of T Cells in 3D ECM Hydrogels

Note that the data presented in this report were acquired over several imaging sessions utilizing evolving (though obviously recorded) variations of the method described below, e.g., using different glass-bottom vessels, different hydrogel volumes, or different cell numbers. For simplicity, the method described here is the one which we found to present the fewest technical problems (e.g., poor hydrogel adherence).

Glass-bottom dishes (MatTek Corporation, Ashland, MA, USA) were first functionalized to enhance adherence of the hydrogel. The glass microwell was first treated with 0.1 N sodium hydroxide for 30 min at room temperature (RT), and subsequently aspirated and air-dried. (3-Aminopropyl)trimethoxysilane (APTMS; Sigma-Aldrich Cat. #281778) was then spread onto the glass microwell and incubated for 3 min at RT, and aspirated before five washes with water for five minutes each on a plate shaker. After aspirating the final wash, 0.5% glutaraldehyde in water was added to the glass microwell and incubated for 1 h at RT. The dish was then washed five times as before, and the microwell was coated with dilute VitroCol solution (500 μg/mL in 0.01 N hydrochloric acid) overnight at 37 °C. After one wash with water and three washes in PBS, the dish was covered with PBS and stored at 4 °C (for no more than 48 h) until use.

To embed T cells in ECM hydrogels, cells at 24 to 48 h post-infection were counted, pelleted, and resuspended in ice-cold serum-free RPMI 1640 at an appropriate cell density. For the experiment shown in Movie S8, uninfected CEM-SS cells were labeled with CellTracker Blue CMAC (Molecular Probes Cat. #C2110) according to manufacturer guidelines, and then mixed with infected CEM-SS cells at a 1:1 ratio prior to centrifugation.

For Matrigel hydrogels, the cell suspension was mixed with Matrigel solution on ice to a final concentration of ~8 mg/mL and final cell density of 2 to 5 million cells/mL (CEM-SS) or 5 to 15 million cells/mL (primary CD4^+^). For collagen hydrogels, ice-cold VitroCol solution was neutralized to pH 7.0 using 0.1 N sodium hydroxide, and 10× PBS was added to achieve a final 1× concentration. The cell suspension was then mixed with the neutralized gel solution to achieve a final collagen concentration of 1.6 mg/mL and cell densities as above. The cold gel (Matrigel or collagen) suspension was then pipetted onto the functionalized glass microwell (300 μL for a 14 mm microwell in a 35 mm dish) and was quickly placed into a 37 °C/5% CO_2_ incubator. After 30 min, 1.7 mL of prewarmed culture medium was carefully pipetted onto the hydrogel, and the dish was placed back in the incubator for 24 h.

### 3.5. Live Cell Time-Lapse Imaging of T Cells Embedded in 3D ECM Hydrogels

The medium covering the 3D ECM hydrogel was aspirated and replaced with prewarmed CO_2_-independent medium supplemented with 10% FBS. The glass-bottom dish was then placed on the microscope stage surrounded by a temperature-controlled enclosure preheated to 37 °C with a beaker of water for humidity. Fluorescence and brightfield images were acquired using 20× or 40× air objectives on a DeltaVision microscope (Applied Precision, Issaquah, WA, USA) on an Olympus IX70 base with a Xenon light source and automated *Z*-axis control, point visitation, and time-lapse acquisition. Using Fiji 2.0.0-rc-41 [52], Z-slices were projected (by maximum intensity for fluorescence images, and by minimum intensity for brightfield images), intensity levels were adjusted, and gamma correction was applied where indicated. Quantification of mean fluorescence intensity and skewness (Figure 3B,C) was performed using Fiji 2.0.0-rc-41 [52] by manually circling each cell at each time point as a region of interest, which was then exported using the Analyze/Measure tool. Any frames where a cell of interest was obscured by another cell were omitted from the analyses. Note that the times shown in Figure 3 do not necessarily correspond to the times in Movies S6 and S7, due to the realignment of the traces as described in the legend for Figure 3, though the same realignment was used for Figure 3B,C. The *in vitro* data shown in Figure 2, Figure 3 and Figure 4 were taken from six 18–24 h imaging sessions and a total of 55 imaging fields.

## 4. Conclusions

HIV-1-induced T cell-based syncytia so far have been considered short-lived artifacts of *in vitro* cell culture. Results presented in this study, however, show that when cultivated in 3D ECM hydrogels, which more closely resemble the environment in lymphoid tissue of infected individuals, HIV-1-infected T lymphocytes can form small syncytia that closely mimic those observed in lymph nodes of humanized mice (and possibly in secondary lymphoid tissue of infected individuals). Using this 3D *in vitro* system allows us also to document that such small T cell-based syncytia can transfer virus to uninfected cells. Further investigations of potential contributions to virus spread and pathogenesis thus are clearly warranted.

## Supplementary Materials

The following are available online at http://www.mdpi.com/1999-4915/7/12/2959/s1, Zenodo DOI:10.5281/zenodo.35385 (https://zenodo.org/record/35385#.VnIyrPmqrtw).

- Movie S1: HIV-1-infected cells in the lymph node of humanized mice.
- Movie S2: Syncytia in the lymph node contact uninfected T cells without undergoing cell-cell fusion.
- Movie S3: CD4^+^ T cells in 3D culture form small syncytia with elongated morphology.
- Movie S4: Small CEM-SS syncytia in 3D culture can dynamically change their morphology.
- Movie S5: CD4^+^ T cells in 3D culture exhibit *in vivo*-like migratory behavior.
- Movie S6: Uninucleated infected cells and syncytia can transfer virus to target cells without fusion.
- Movie S7: A virus transfer event between a syncytium and two uninfected target cells.
- Movie S8: Cell-to-cell transfer of virus can take place while cells are migrating.
- Movie S9: Migrating infected cells can deposit a trail of released virus particles.
