# Supplementary material for: HIV-1-Induced Small T Cell Syncytia Can Transfer Virus Particles to Target Cells through Transient Contacts
Source: Viruses. 2015 Dec 12;7(12):6590–603. doi: 10.3390/v7122959 (PMC4690882; doi:10.3390/v7122959)
Supplement: Supplementary file 1 [file viruses-07-02959-s001.pdf]

Supplementary Materials: HIV-1-Induced Small T Cell Syncytia Can Transfer Virus Particles to Target Cells through Transient Contacts*

Menelaos Symeonides, Thomas T. Murooka, Lauren N. Bellfy, Nathan H. Roy, Thorsten R. Mempel and Markus Thali

* Zenodo DOI: 10.5281/zenodo.35385; Available on: https://zenodo.org/record/35385#.VnIyrPmqrtw

Supplementary Movie Legends (Movies S1–S9)

Movie S1. HIV-1-infected cells in the lymph node of humanized mice.

(Available on: https://zenodo.org/record/35385/files/Movie_S1.mp4)

Humanized BLT mice were injected in the footpad with HIV-nGFP, where GFP is highly enriched in cellular nuclei, and the draining popliteal lymph node prepared for MP-IVM at day 2. Representative infected cells (GFP^+; green) that display one, two or three discernible nuclei are shown (**top**). In the bottom panels, green fluorescence signals above 80% of the intensity maximum were used to define cell nuclei, which are shown in white. The syncytium with two discernible nuclei remains elongated throughout the recording, while the syncytium with three discernible nuclei switches between coordinated and uncoordinated motility. Each individual frame is a maximum intensity projection of 11 *z*-stacks spaced 4 µm apart (for a total volume of 40 µm). Time is shown in minutes and seconds. Scale bar = 20 µm. See also Figure 1A.

Movie S2. Syncytia in the lymph node contact uninfected T cells without undergoing cell-cell fusion.

(Available on: https://zenodo.org/record/35385/files/Movie_S2.mp4)

In vitro-generated central memory $CD4^+$ T cells, either infected with HIV-GFP (GFP^+; green) or uninfected (labeled with CellTracker Orange; red), were adoptively transferred by footpad injection into BLT mice pretreated with antiretroviral drugs (100 mg/kg FTC, 150 mg/kg TDF). After 12 h, the draining popliteal lymph node was prepared for MP-IVM. Two representative movies of T cell migration prior to (yellow circle) and during (blue circle) transient interactions with syncytia are shown, demonstrating cellular interactions without fusion. Each individual frame is a maximum intensity projection of 11 *z*-stacks spaced 4 µm apart (for a total volume of 40 µm). Time is shown in minutes and seconds. Scale bar = 40 µm. See also Figure 1F,G.

Movie S3. $CD4^+$ T cells in 3D culture form small syncytia with elongated morphology.

(Available on: https://zenodo.org/record/35385/files/Movie_S3.mp4)

Primary human $CD4^+$ T cells isolated from a healthy donor were infected with VSV-G-pseudotyped NL4-3$^{Gag\text{-}iGFP}$ virus. The next day, cells were embedded in a 3D collagen gel as described in the Experimental Section, and 12 h later imaged live at 37 °C using a 20× objective on a DeltaVision widefield microscope. Six 3 µm-spaced Z-slices were taken every 20 s, and were subsequently projected into one image. The syncytium seen here in green has two nuclei located at opposite ends, and a central bulged region with high amounts of viral Gag. The diffuse fluorescence is a result of this syncytium being located at a higher part of the gel, where the limitations of widefield imaging become more prominent. See also Figure 2A.

Movie S4. Small CEM-SS syncytia in 3D culture can dynamically change their morphology.

(Available on: https://zenodo.org/record/35385/files/Movie_S4.mp4)

CEM-SS cells were infected with VSV-G-pseudotyped NL4-3$^{Gag\text{-}iGFP}$ virus. The next day, cells were embedded in a 3D Matrigel gel as described in the Experimental Section, and 24 h later imaged live at 37 °C using a 40× objective on a DeltaVision widefield microscope. Seven 2 µm-spaced Z-slices were taken every 5 min, and were subsequently projected into one image. A syncytium with two nuclei (dark areas within the cell in the GFP

channel) begins with two lobes, which merge into a coordinated round morphology as the cell begins to migrate through the gel and leaves the plane of focus. See also Figure 2B.

Movie S5. $CD4^+$ T cells in 3D culture exhibit *in vivo*-like migratory behavior.

(Available on: https://zenodo.org/record/35385/files/Movie_S5.mp4)

Primary human $CD4^+$ T cells were infected, embedded in collagen, and imaged as in Movie S3 (though with a 10 s time lapse). The uninucleated infected cell migrating across the field moves by 108 μm over 560 s, for a mean velocity of 11.58 μm/min. Such fast directed amoeboid motility is not typically observed in classical 2D culture and requires the presence of a 3D ECM. See also Figure 2C.

Movie S6. Uninucleated infected cells and syncytia can transfer virus to target cells without fusion.

(Available on: https://zenodo.org/record/35385/files/Movie_S6.mp4)

CEM-SS cells were infected, embedded in Matrigel, and imaged as in Movie S4. Virus transfer from a uninucleated infected cell (top) and a syncytium (bottom left) to a number of target cells (denoted by T) can be seen here. The uninucleated infected cell transfers virus to target cells T1–T5, and the syncytium transfers virus to target cells T6–T7. Images shown represent brightfield in gray (bottom right), or Gag-iGFP in green, shown either with normal scaling (bottom left, and merged with brightfield at top right), or with a 0.6 gamma correction applied and enhanced scaling to better show appearance of Gag-iGFP puncta on target cells (top left). Scale bar = 30 μm. A yellow arrow indicates the moment where the uninucleated infected cell begins transferring virus to target cells T1–T3, and all of the other transfer events in this field are happening at roughly the same time. At this time, Gag-iGFP puncta appear to distribute between cells T1–T3 in a progressive fashion, beginning from the point of contact with the infected cell (see also Figure 3A, middle panel). Cells T4 and T5 also receive virus particles from this infected cell, and T4 can be seen migrating away at the end. Also note a trail of released virus left behind by the uninucleated infected cell as it migrates from left to right from 08:00:00 to 09:10:00 (see also Movies S7 and S9, Figure 4 for similar events in syncytia). The syncytium's targets, T6 and T7, are already in intimate contact with it at the start of the movie, and are obscured by lobes of the syncytium. At the 10:45:00 mark, cell T6 breaks free from the syncytium, now harboring a large amount of virus particles on its surface, as the syncytium slowly migrates away, and cell T7 also appears to harbor virus particles on its surface by the final time point.

Movie S7. A virus transfer event between a syncytium and two uninfected target cells.

(Available on: https://zenodo.org/record/35385/files/Movie_S7.mp4)

CEM-SS cells were infected, embedded in Matrigel, and imaged as in Movies S4 and S6. The syncytium shown here initially has two nuclei, but soon fuses with an uninfected cell and now has three clearly visible nuclei. It remains stationary for several hours, before beginning to migrate towards a pair of uninfected target cells (top). Very soon after contact, virus particles can be seen covering the surface of both cells, one of which eventually migrates away. See also Figure 3A (left panel). Note also another instance of a dense accumulation of cell-free virus particles in what appears to be a pocket within the hydrogel that the syncytium moves one of its lobes out of, revealing the deposited free virus, before it moves back into the pocket. See also Figure 4A.

Movie S8. Cell-to-cell transfer of virus can take place while cells are migrating.

(Available on: https://zenodo.org/record/35385/files/Movie_S8.mp4)

CEM-SS cells were infected, later mixed with CMAC-labeled uninfected CEM-SS cells (shown in blue), embedded in collagen, and imaged as above. A syncytium with two nuclei (one of which bears CMAC signal, indicating that it formed recently and not before the infected culture was mixed with the labeled uninfected cells) migrates across the field. Its trailing edge contacts an uninfected CMAC-labeled cell, which is then dragged along with it, and finally dropped in the corner of the field. The target cell now bears virus particles on its surface, and is no longer in contact with the syncytium, which has moved into a different focal plane and stopped migrating (not shown). The image was refocused at the 10:30:00 mark to better show the target cell and the virus particles on its surface. Note that this movie also shows an instance of newly synthesized Gag-iGFP appearing in a previously uninfected cell (not the target cell contacted by the syncytium). This non-CMAC

labeled uninfected cell appears in the bottom left of the field at 07:20:00 and exhibits steadily increasing diffuse intracellular signal, as documented in Figure 3A (right) and Figure 3B,C (green traces).

Movie S9. Migrating infected cells can deposit a trail of released virus particles.

(Available on: https://zenodo.org/record/35385/files/Movie_S9.mp4)

CEM-SS cells were infected, embedded in Matrigel, and imaged as in Movies S4, S6, and S7. A syncytium with 3 nuclei switches into a coordinated morphology and begins migrating across the field. Released virus particles can be seen in its wake (also shown enlarged and with increased brightness as an inset). Shortly after the end of the movie, the cell-free virus accumulation appeared to dissipate (not shown), but it could not be determined whether this was because of photobleaching or if they had in fact diffused away. See also Figure 4B.

© 2015 by the authors; licensee MDPI, Basel, Switzerland. This article is an open access article distributed under the terms and conditions of the Creative Commons by Attribution (CC-BY) license (http://creativecommons.org/licenses/by/4.0/).
